# Supplementary figures and images for: Spatial chromatin accessibility sequencing resolves high-order spatial interactions of epigenomic markers
Source: eLife. 2024 Jan 18;12:RP87868. doi: 10.7554/eLife.87868 (PMC10945591; doi:10.7554/eLife.87868)

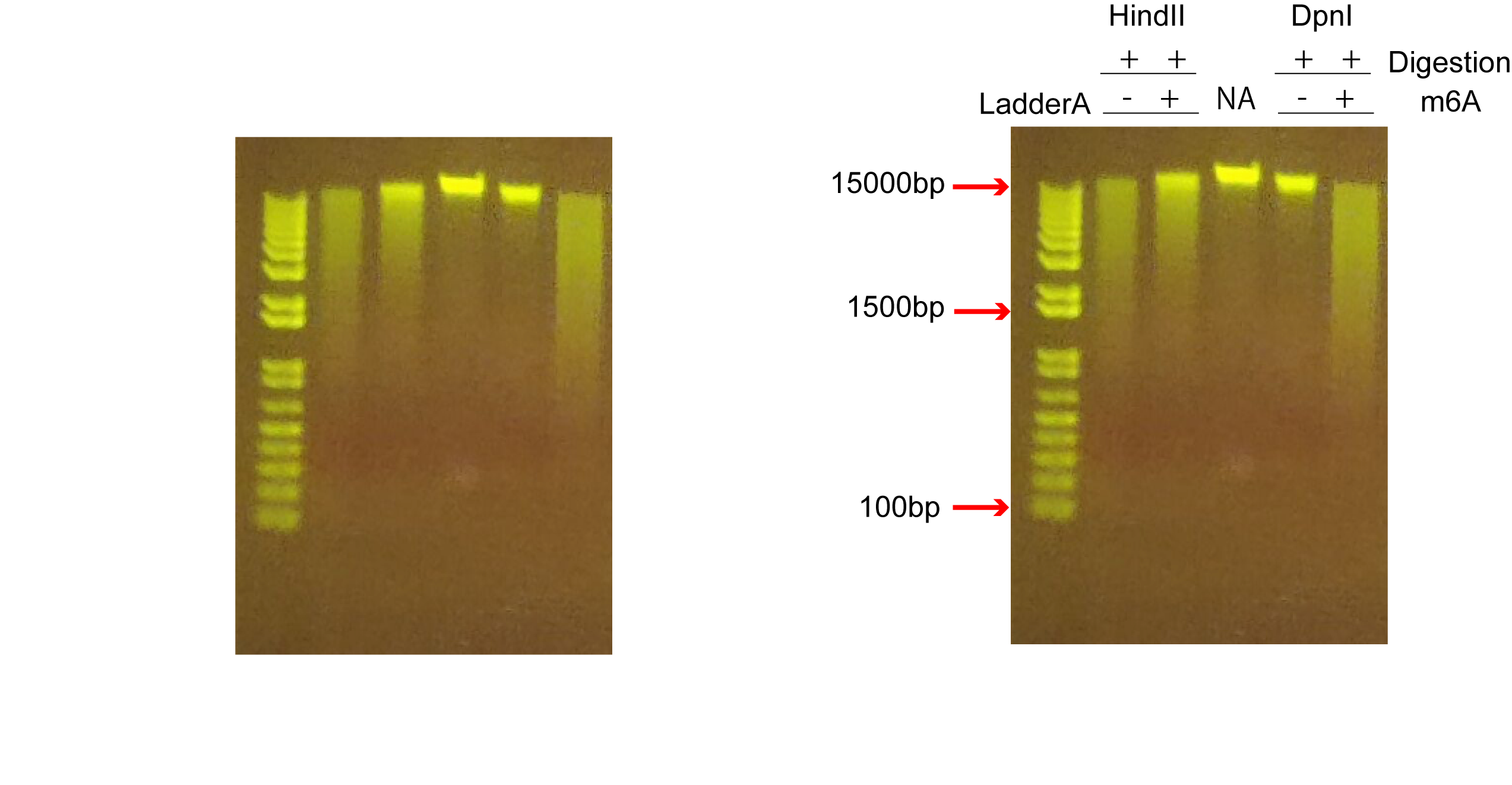

Supplement: Figure 1—figure supplement 1—source data 1. [file elife-87868-fig1-figsupp1-data1.zip › Figure 1-figure supplement 1-source data 1/Fig1-1.source.20231221.a.tif]

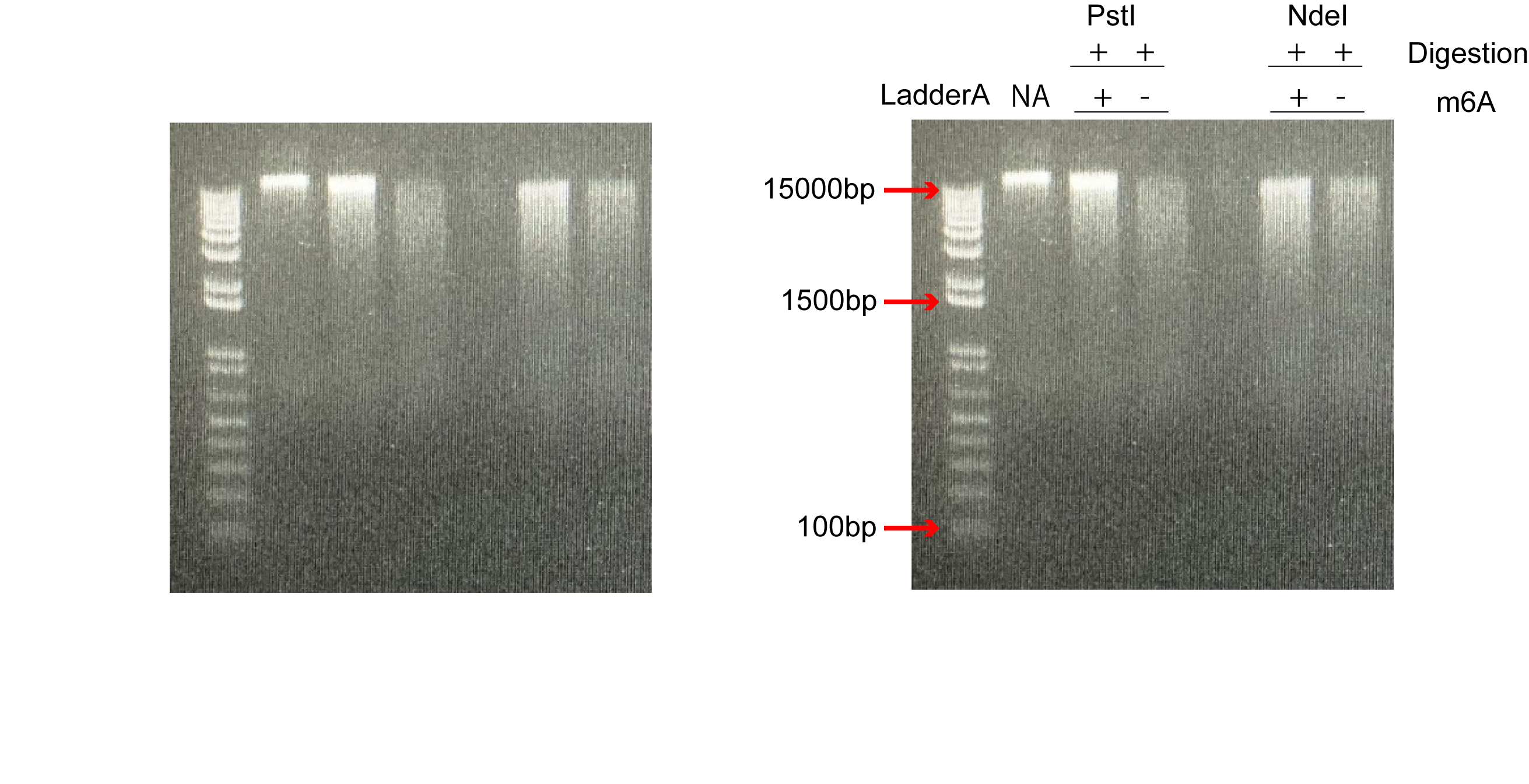

Supplement: Figure 1—figure supplement 1—source data 1. [file elife-87868-fig1-figsupp1-data1.zip › Figure 1-figure supplement 1-source data 1/Fig1-1.source.20231221.b.tif]

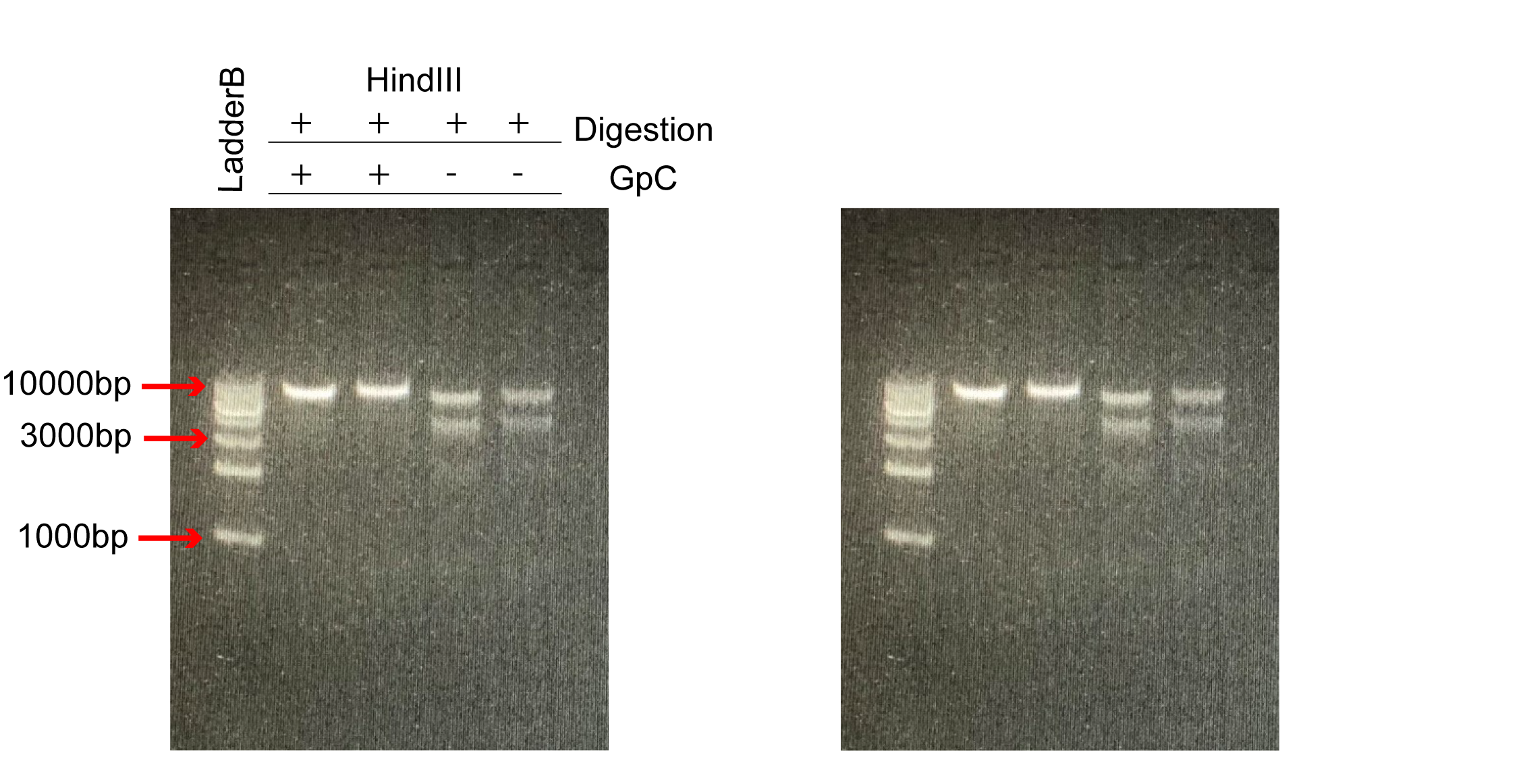

Supplement: Figure 1—figure supplement 1—source data 1. [file elife-87868-fig1-figsupp1-data1.zip › Figure 1-figure supplement 1-source data 1/Fig1-1.source.20231221.c.tif]

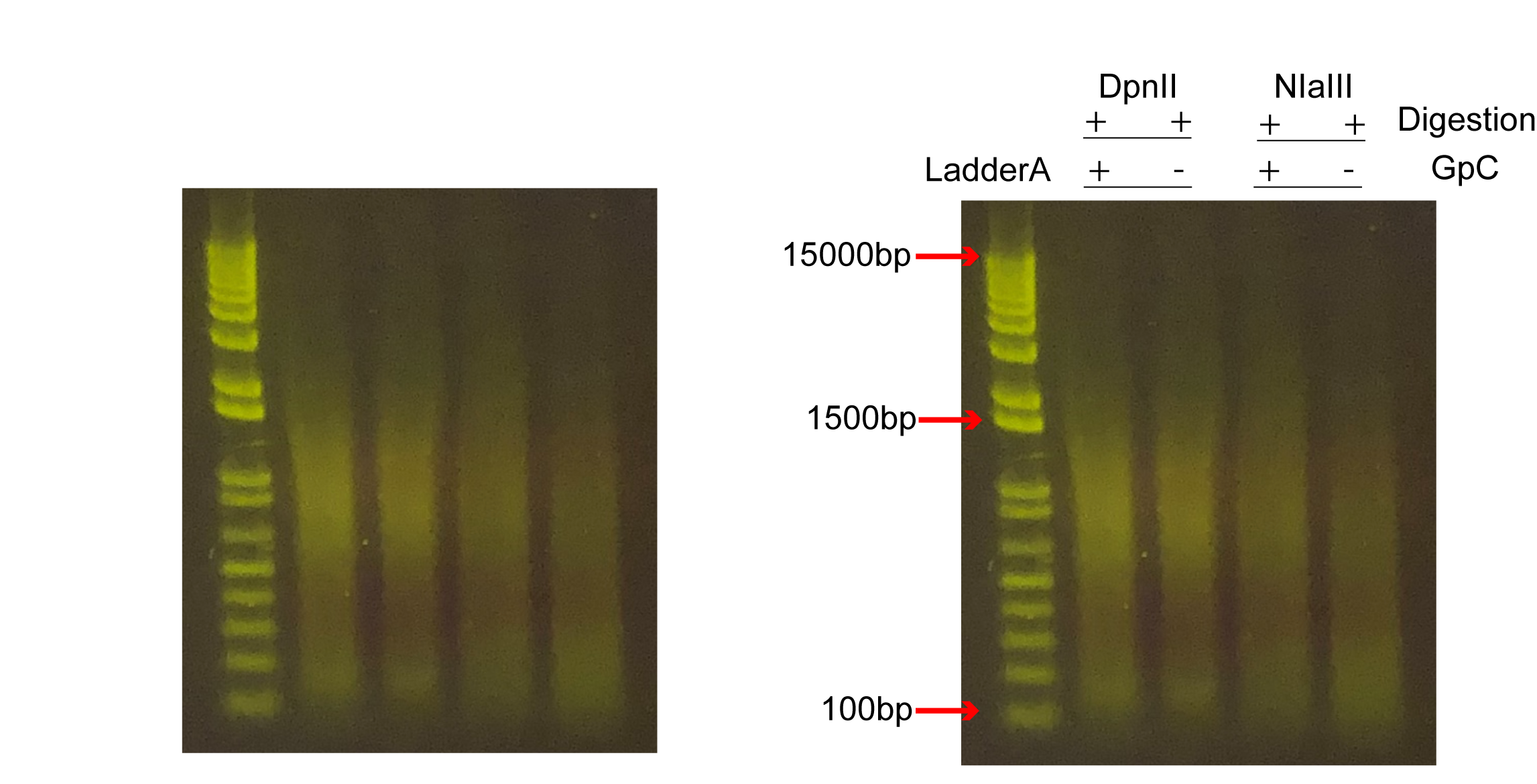

Supplement: Figure 1—figure supplement 1—source data 1. [file elife-87868-fig1-figsupp1-data1.zip › Figure 1-figure supplement 1-source data 1/Fig1-1.source.20231221.d.tif]

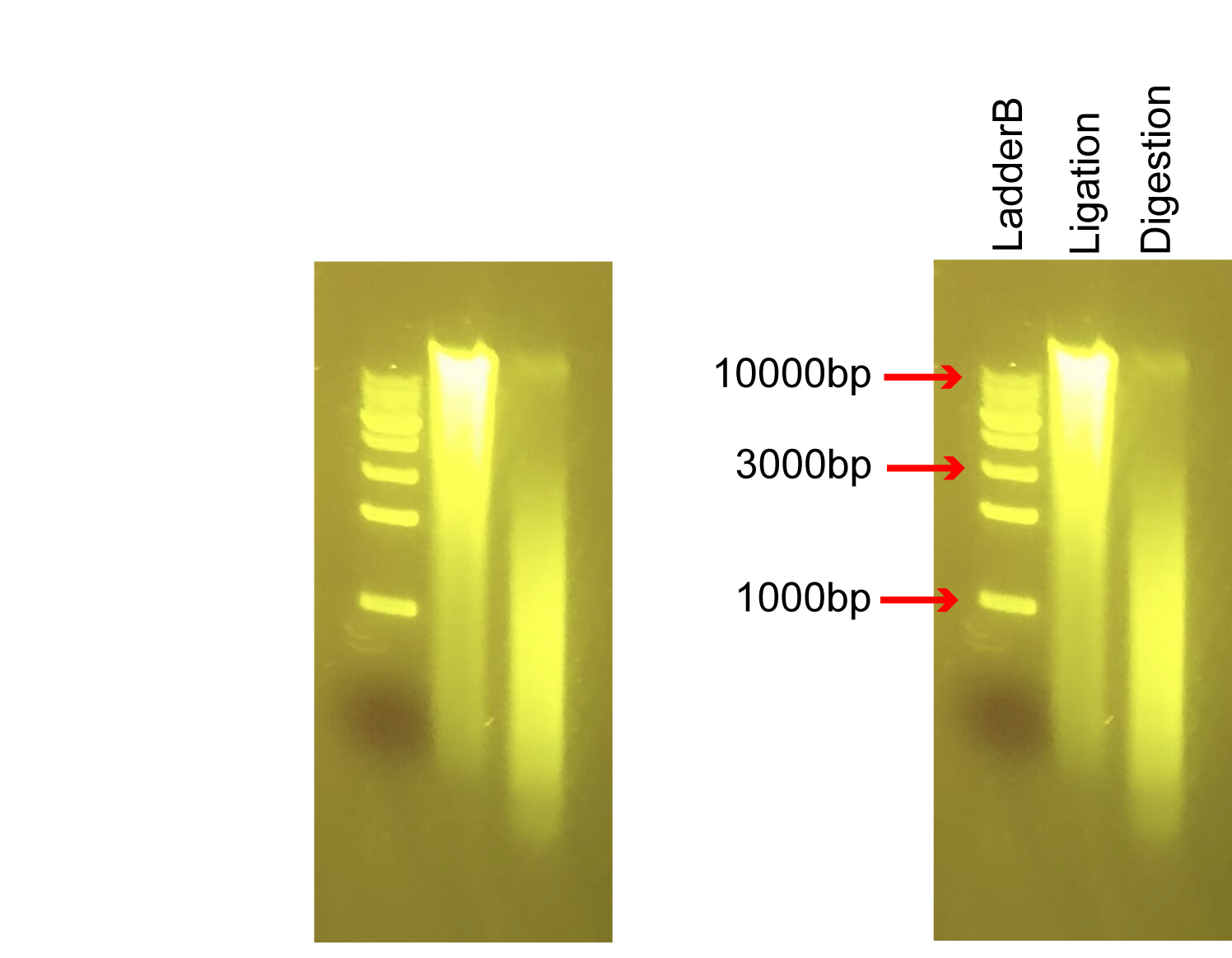

Supplement: Figure 1—figure supplement 1—source data 1. [file elife-87868-fig1-figsupp1-data1.zip › Figure 1-figure supplement 1-source data 1/Fig1-1.source.20231221.e.tif]
